# Implementation and preliminary evaluation of a practical digital workflow for routine laser‐radiation field coincidence quality assurance on the CyberKnife M6

**DOI:** 10.1002/pro6.70103

**Published:** 2026-10-24

**Authors:** Linzheng Hu, Yongkang Zhao, Chunling Zheng, Jiajun Cai, Yajun Jia, Yi Tao, Yikun Liu, Yin Zhang, Zhitao Dai

**Affiliations:** ^1^ National Cancer Center/National Clinical Research Center for Cancer/Cancer Hospital and Shenzhen Hospital Chinese Academy of Medical Sciences and Peking Union Medical College Shenzhen China; ^2^ School of Computer Science and Artificial Intelligence Wuhan University of Technology Wuhan China

**Keywords:** CyberKnife, digital imaging, laser‐radiation field coincidence, QA StereoChecker, quality assurance

## Abstract

**Purpose:**

To implement and preliminarily evaluate a practical digital acquisition‐and‐analysis workflow for routine laser‐radiation field coincidence quality assurance (QA) on the CyberKnife M6 by integrating multileaf collimator (MLC) QA tool‐based laser reference definition, QA StereoChecker (QASC)‐based external digital image acquisition, and automated analysis with CKQA, an in‐house CyberKnife QA software, and to compare this workflow with clinically realistic film‐based alternatives.

**Methods:**

Methods a and b were film‐based measurements performed at the perch position. Method a used manual laser marking on film, whereas method b used the central metal marker of the MLC QA tool to define the laser reference. Method c used QASC as an external digital image‐acquisition platform in combination with the MLC QA tool, and method d used film on the same QASC‐based setup as a control. All methods were tested with 10 fixed collimators (5–40 mm). Images were analyzed using ImageJ and CKQA. Mean deviation, standard deviation, root mean square error (RMSE), and pairwise two‐sided exact Wilcoxon signed‐rank tests were used for exploratory comparisons.

**Results:**

All evaluated workflows achieved submillimeter mean deviations. Across both analysis approaches, the mean deviation ranked consistently in descending order: method a > method b > method d > method c. Under the present experimental conditions, method c yielded the lowest mean deviation and RMSE (0.116 mm and 0.155 mm with CKQA), whereas method a showed the largest deviations. Dataset‐level exact Wilcoxon signed‐rank tests showed that several comparisons involving method a were statistically significant, but statistically significant differences were not uniform across both ImageJ‐ and CKQA‐based comparisons. For comparisons between analysis methods within each workflow, CKQA and ImageJ were not significantly different for methods a and b, whereas CKQA yielded significantly lower deviations than ImageJ for methods c and d (*p* < 0.05).

**Conclusions:**

Laser‐radiation field coincidence testing is an established CyberKnife QA procedure. The present study implemented and preliminarily evaluated a practical digital acquisition‐and‐analysis workflow for this routine task. The workflow combining MLC QA tool‐based laser reference definition, QASC‐based external digital image acquisition, and automated CKQA analysis provided a standardized and less operator‐dependent procedure under the present experimental conditions. Its main value lies in reducing manual marking, film handling, scanning, and subjective image‐analysis steps rather than in introducing a new physical measurement principle. In centers where QASC is not available, the film‐based workflow using the MLC QA tool (method b) may serve as a practical alternative.

## Graphical abstract

An MLC QA tool–QASC–CKQA workflow achieved the lowest laser‐radiation field coincidence deviations on CyberKnife M6, supporting a simpler, more reproducible routine QA strategy. When QASC is unavailable, the film‐based MLC QA tool workflow provides a practical alternative.

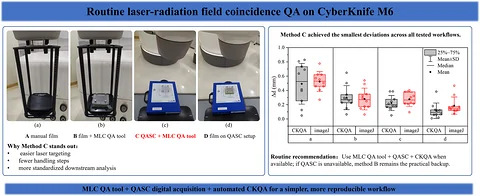

## 1 INTRODUCTION

CyberKnife is a robotic radiosurgery platform that combines image guidance, a compact linear accelerator, and highly noncoplanar beam delivery to achieve submillimeter geometric accuracy in stereotactic radiosurgery (SRS) and stereotactic body radiotherapy (SBRT).^1^, ^2^, ^3^, ^4^, ^5^ As stereotactic techniques continue to expand, the geometric integrity of the treatment system becomes increasingly important, and a rigorous, reproducible quality assurance (QA) program is essential for treatment precision and patient safety.^3^, ^4^, ^5^

Within the CyberKnife geometric QA chain, coincidence between the central‐axis laser and the radiation beam central axis is a fundamental indicator. TG‐135 and TG‐135.B emphasize that both primary and secondary path calibration depend on the geometric consistency between the central‐axis laser and the beam central axis; drift of the laser system can therefore propagate into systematic calibration errors and degrade overall treatment accuracy.^4^, ^5^ The Chinese practical guideline for robotic stereotactic radiotherapy systems, the national standard WS 667–2019, and the Canadian Partnership for Quality Radiotherapy (CPQR) technical quality control guideline likewise include laser‐related checks within routine daily, monthly, or periodic QA workflows.^6^, ^7^, ^8^ Recent commissioning and technical QA data for the CyberKnife S7 further show that submillimeter laser‐beam alignment remains a sensitive and clinically relevant performance metric across platform generations.^9^

Conventional laser‐radiation field coincidence testing is commonly performed with film or mechanical fixtures. Accuray's Physics Essential Guide describes a manual film‐based workflow in detail.^10^ In routine clinical practice, however, this workflow contains several potential sources of geometric uncertainty. First, the manually created reference mark on the film may not form a geometrically regular point, and visual alignment of the laser with the mark can be hindered by laser glare. Second, during alignment, inadvertent pressure on the film may shift the mark position, and subsequent fixation of the film to the holder with tape can introduce additional displacement. Third, after irradiation, flatbed scanning adds another handling step and may contribute additional uncertainty and workflow burden. In addition, ImageJ‐based analysis, although flexible, does not inherently enforce standardized parameter settings, so threshold selection and center identification may remain operator dependent.^10^, ^11^, ^12^, ^13^, ^14^ Collectively, these factors may affect repeatability and efficiency in a QA task that is performed repeatedly in clinical practice. Digital and filmless QA concepts are not new in radiotherapy. CyberKnife‐specific digital QA approaches, such as beam‐path constancy monitoring, radioluminescence imaging, and scintillation or Cherenkov imaging‐based beam shape verification, have been reported previously.^15^, ^16^, ^17^ In conventional linear accelerator platforms, EPID‐based or other digital imaging workflows have long been used for light‐radiation field congruence testing and may improve efficiency, standardization, and repeatability compared with film‐based approaches.^18^, ^19^, ^20^, ^21^, ^22^ The recently published AAPM TG report 330 provides comprehensive guidance on EPID‐based linac QA,^23^ and dedicated semi‐automatic software has also been developed for LINAC light‐radiation field coincidence analysis.^24^ In the present study, QA StereoChecker (QASC) was not treated as an onboard EPID; rather, it was used as an external digital image‐acquisition platform whose methodological role is analogous to that of EPID‐based digital workflows, while the actual deviation measurement was completed using ImageJ or an in‐house automated analysis software (CKQA).^25^

The present study was designed as a workflow implementation and preliminary validation study rather than as the introduction of a new physical measurement principle. Against this background, we evaluated four clinically realistic laser‐radiation field coincidence workflows and two image‐analysis approaches on a CyberKnife M6. The aim was to determine whether the integrated workflow of multileaf collimator (MLC) QA tool‐based laser reference definition, QASC‐based external digital acquisition, and automated CKQA analysis could provide a more standardized and less operator‐dependent option for routine clinical QA. We also assessed whether the film‐based MLC QA tool workflow (method b) could serve as a practical alternative in centers without QASC.

## 2 METHODS

### 2.1 Study design

This study compared four measurement workflows and two image‐analysis methods to support selection of a routine clinical workflow for CyberKnife laser‐radiation field coincidence testing. The measurements were performed on an Accuray CyberKnife M6 using 10 fixed circular collimators with diameters of 5, 7.5, 10, 12.5, 15, 20, 25, 30, 35, and 40 mm. For each measurement workflow, images were analyzed with ImageJ and with the in‐house CKQA software, yielding eight datasets in total. All measurements were acquired under the same irradiation conditions, and the source‐to‐detector distance (SDD) or source‐to‐film distance was kept at 800 mm whenever applicable. The main analysis was restricted to fixed circular collimators.

The two measurement positions were selected according to the practical implementation of the corresponding clinical QA workflows. The conventional film‐based workflows were performed at the perch position because this is the routine position used for laser‐radiation field coincidence testing with the birdcage assembly, and the film mount fixture is designed to position the film plane at an SDD of 800 mm when attached to the birdcage. In contrast, QASC is a relatively heavy external digital image‐acquisition device and cannot be safely or reproducibly mounted onto the birdcage assembly using the available fixtures. Therefore, the QASC‐based workflows were performed on the treatment couch at the DailyQA node. To reduce geometric differences between the two setups, treatment head alignment was checked using the pinhole laser collimator before measurement, and the measurement plane was set to an SDD of 800 mm for both Perch‐based and couch‐based workflows.

### 2.2 Principle of laser‐radiation field coincidence measurement

The central‐axis laser in the CyberKnife system indicates the nominal position of the radiation beam central axis. Laser‐radiation field coincidence was evaluated by identifying the laser reference point and the radiation field center on each image and then calculating the Euclidean distance between them. If the laser point is denoted as (x_0_, y_0_) and the radiation field center as (x_i_, y_i_), the deviation *Δd* is defined as $\Delta d=\sqrt{{\left(x_{0}-x_{i}\right)}^{2}+{\left(y_{0}-y_{i}\right)}^{2}}$. Smaller values indicate better geometric coincidence.^4^, ^5^, ^6^, ^7^

### 2.3 Measurement workflows

The experimental setup and positioning for the four measurement workflows are shown in Figure 1. Figure 1 illustrates the perch‐based film workflows for methods a and b and the QASC‐based couch workflows for methods c and d.

**FIGURE 1:**
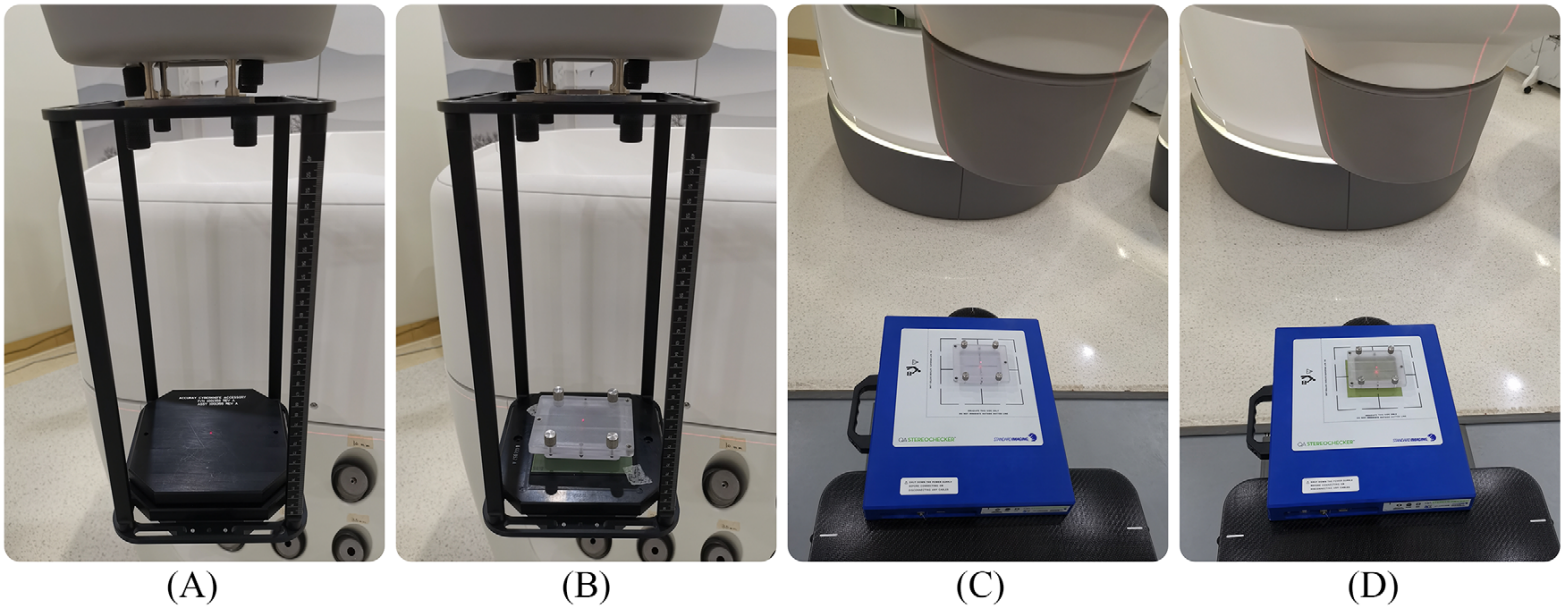
Experimental setup and positioning for the four laser‐radiation field coincidence measurement workflows (methods a‐d).

#### 2.3.1 Method a: Manual film marking at perch position

Method a (manual film marking at perch position): This workflow followed the conventional film‐based approach described in the Physics Essential Guide.^10^ The robotic arm was positioned at perch, and film was fixed to the birdcage film holder at an SDD of 800 mm. The central‐axis laser point was manually marked on the film surface, a 1.5‐cm buildup layer was added, and 2000 MU was delivered for each collimator. The films were scanned at high resolution and exported in TIFF format for subsequent analysis. This workflow involved manual laser‐to‐marker alignment, film fixation, and postirradiation scanning before image analysis.

#### 2.3.2 Method b: Film with the MLC QA tool at perch position

Method b (film with MLC QA tool at perch position): The setup was identical to that of method a except that no manual mark was made on the film. Instead, the MLC QA tool was placed over the film, and the central metal marker of the tool was aligned with the central‐axis laser. When the aligned marker was irradiated, its attenuation created a faint low‐density spot near the center of the exposed film, and the center of this spot was taken as the laser reference point for coincidence measurement. The reflective metal tip also provided a sharper visual target than a hand‐drawn film mark, potentially reducing alignment ambiguity. The same irradiation and scanning workflow was then applied.

#### 2.3.3 Method c: Direct digital acquisition with QASC

Method c (direct digital acquisition with QASC): QASC was positioned on the treatment couch with the robotic arm at the DailyQA position. The couch was adjusted so that the measurement plane was located at an SDD of 800 mm. The MLC QA tool was placed on top of QASC and aligned so that its central metal marker coincided with the central‐axis laser. In practice, the strong specular reflection at the metal tip facilitated visual confirmation of laser coincidence, while the rigid setup allowed alignment by gentle adjustment of the tool rather than direct pressure on a film sheet. Images were acquired directly with the QASC software after delivery of 2000 MU irradiation for each collimator, without film development or scanning.

#### 2.3.4 Method d: Film on the QASC‐based setup

Method d (film on the QASC‐based setup): This workflow used the same couch setup, SDD, and field conditions as method c, but film was placed in the central region of the MLC QA tool on top of QASC. The irradiated films were then processed and scanned in the same manner as methods a and b. Method d served as a control to isolate the effect of image‐acquisition medium under nearly identical geometry and alignment conditions.

### 2.4 Image analysis

#### 2.4.1 ImageJ workflow

ImageJ analysis: Film TIFF files and QASC‐acquired images were imported into ImageJ. The pixel size was calibrated to physical distance. For film images, the red channel was used after channel separation to improve radiation field contrast.^10^, ^12^, ^13^, ^14^, ^26^ The laser point was identified, the radiation field was segmented by thresholding, and the field center was determined from the extracted region. The laser‐radiation field deviation was then calculated according to the definition above.

#### 2.4.2 CKQA workflow

CKQA analysis: The same images were analyzed with an in‐house automated software package (CKQA). The workflow included image import, grayscale conversion, thresholding, denoising with Gaussian and median filters, connected‐component analysis of the radiation field, automatic identification of the laser reference point (the manual film mark for method a or the metal‐marker spot for methods b‐d), and automated calculation of the final deviation. The major CKQA workflow is summarized in Figure 2, and representative stepwise processing results are shown in Figure 3, which presents the complete processing sequence from raw image acquisition to beam and laser center detection, with the 10‐mm scale bar providing a physical reference for the submillimeter deviations reported in this study. By standardizing region selection and center determination, CKQA was designed to reduce operator dependence.

**FIGURE 2:**
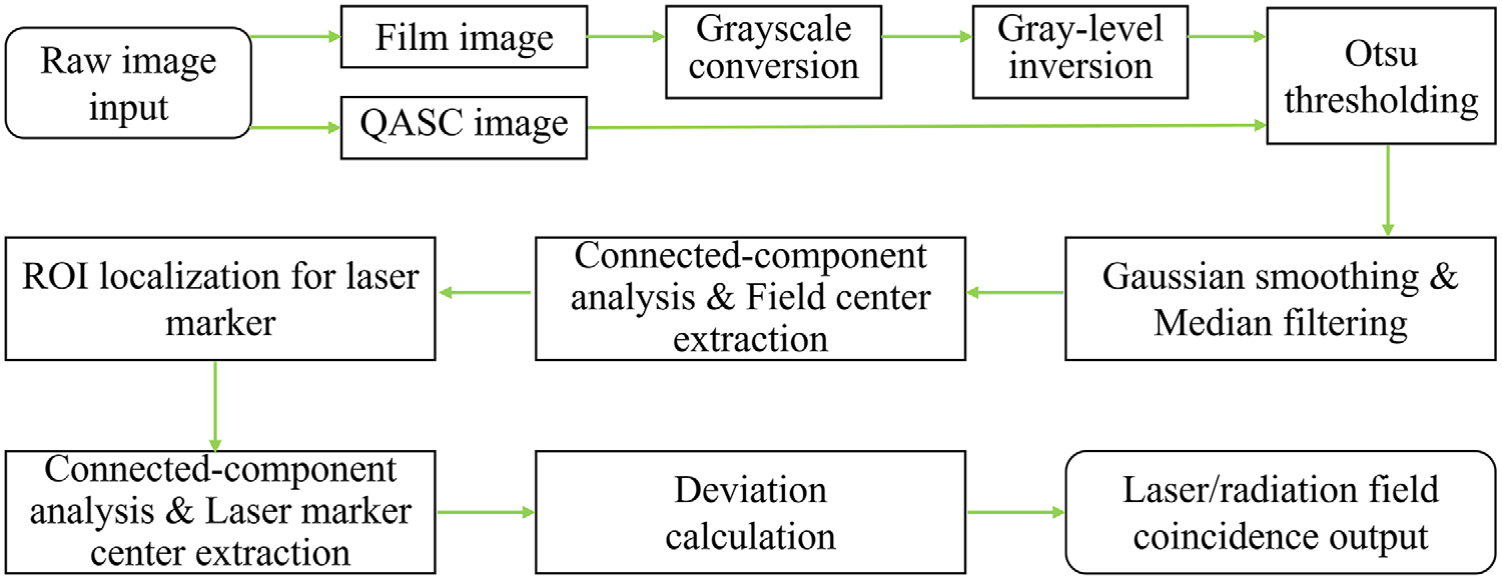
Workflow of the CKQA software for automated analysis of laser‐radiation field coincidence.

**FIGURE 3:**
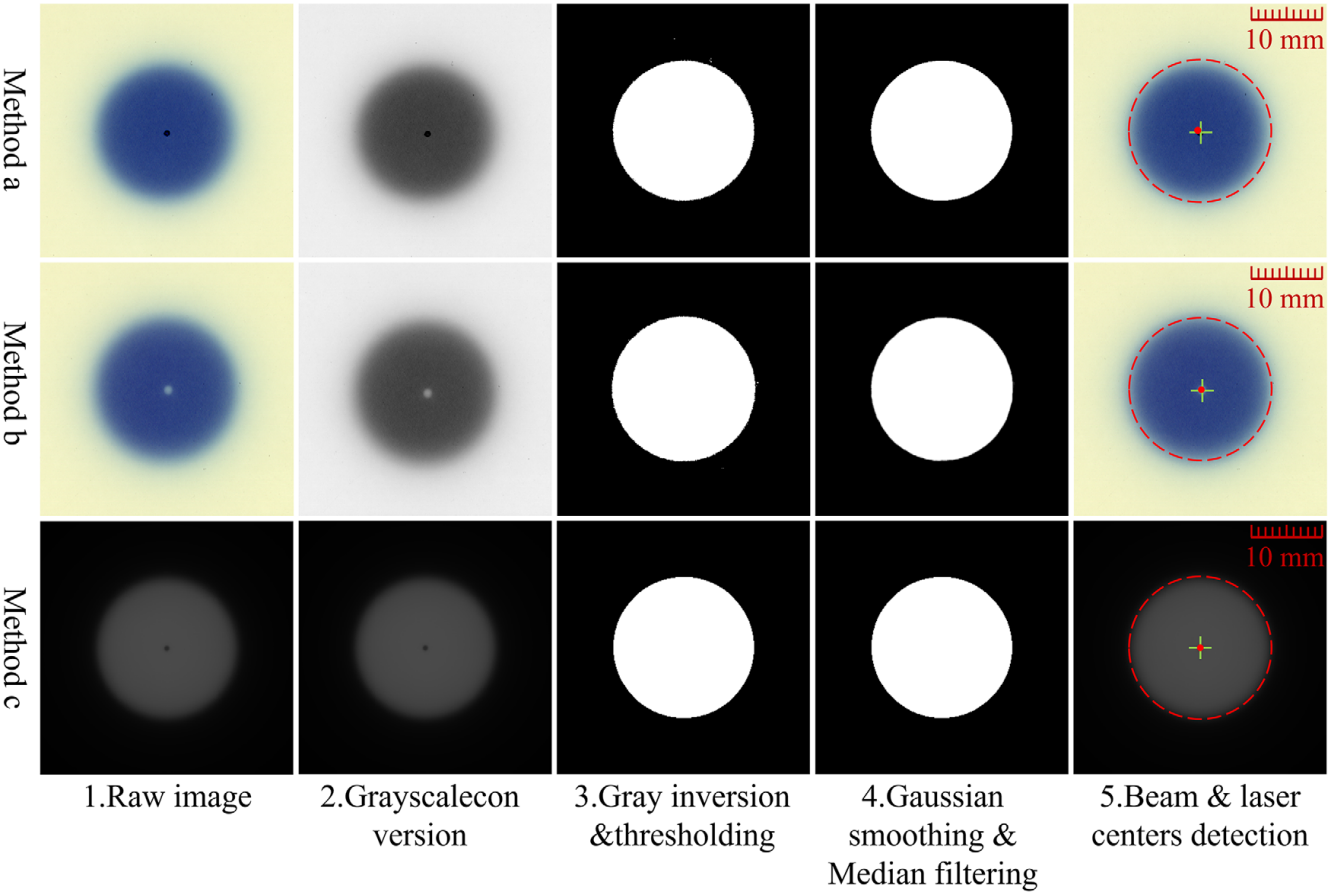
Representative stepwise CKQA processing results for automated laser‐radiation field coincidence analysis. A 10‐mm physical scale bar with millimeter tick marks is shown in each row to indicate the spatial scale of the cropped images.

CKQA was used in this study as an in‐house automated image‐analysis tool to standardize image preprocessing, center extraction, and deviation calculation. However, independent mathematical validation of the center‐finding algorithm using synthetic digital phantom images with predefined sub‐pixel offsets was not included in the present workflow evaluation.

### 2.5 Statistical analysis

For each of the eight datasets, the mean, median, maximum, minimum, standard deviation, and root mean square error (RMSE) were calculated. Because the sample size was small and normality could not be assumed, paired comparisons were performed using the two‐sided exact Wilcoxon signed‐rank test. Statistical analyses were recalculated using Python 3.13.5 with scipy.stats.wilcoxon in SciPy 1.17.0. Pairwise tests were considered exploratory because of the small sample size and the number of comparisons; therefore, no formal adjustment for multiple comparisons was applied, and P values should be interpreted cautiously. Boxplots were used to visualize data dispersion.

## 3 RESULTS

### 3.1 Comparison among measurement methods

Table 1 summarizes the laser‐radiation field coincidence deviations obtained with the four measurement methods and the two analysis workflows, Figure 4 shows the distribution of the eight datasets, and Figure 5 summarizes the dataset‐level pairwise exact Wilcoxon signed‐rank test results. Across both ImageJ and CKQA, the mean deviation followed the same descending order: method a > method b > method d > method c. Method a showed the largest mean deviation (0.525 mm with ImageJ and 0.494 mm with CKQA), whereas method c showed the smallest mean deviation (0.189 mm with ImageJ and 0.116 mm with CKQA). RMSE showed the same pattern; the lowest RMSE was observed for method c (0.220 mm with ImageJ and 0.155 mm with CKQA). All mean deviations were below the commonly used 1.0‐mm clinical tolerance. Dataset‐level pairwise comparisons showed that several comparisons involving method a were statistically significant, particularly when method a was compared with methods b, c, or d. Method c with CKQA also showed significantly lower deviations than several other datasets, including b‐ImageJ, b‐CKQA, c‐ImageJ, and d‐ImageJ. However, significant differences were not consistent between both ImageJ and CKQA analyses; for example, b‐ImageJ versus c‐ImageJ and c‐ImageJ versus d‐ImageJ were not statistically significant. Therefore, Figure 5 should be interpreted as an exploratory dataset‐level comparison matrix; broader method‐level conclusions should be drawn cautiously.

**TABLE 1:** Laser‐radiation field coincidence deviations (mm) obtained with the four measurement workflows and the two analysis methods.

Collimator diameter (mm)	a		b		c		d	
	ImageJ	CKQA	ImageJ	CKQA	ImageJ	CKQA	ImageJ	CKQA
5	0.776	0.691	0.177	0.281	0.121	0.063	0.227	0.187
7.5	0.554	0.636	0.349	0.341	0.174	0.058	0.306	0.145
10	0.572	0.733	0.248	0.294	0.074	0.120	0.211	0.178
12.5	0.622	0.829	0.540	0.648	0.115	0.021	0.390	0.246
15	0.625	0.808	0.063	0.151	0.143	0.129	0.323	0.387
20	0.407	0.415	0.324	0.260	0.126	0.009	0.402	0.387
25	0.459	0.395	0.294	0.231	0.155	0.122	0.215	0.167
30	0.504	0.126	0.490	0.503	0.467	0.384	0.095	0.051
35	0.270	0.255	0.147	0.226	0.171	0.067	0.216	0.214
40	0.457	0.048	0.172	0.065	0.338	0.181	0.381	0.277
Mean	0.525	0.494	0.280	0.300	0.189	0.116	0.277	0.224
Median	0.529	0.525	0.271	0.271	0.149	0.094	0.267	0.200
Maximum	0.776	0.829	0.540	0.648	0.467	0.384	0.402	0.387
Minimum	0.270	0.048	0.063	0.065	0.074	0.009	0.095	0.051
SD	0.132	0.271	0.144	0.159	0.114	0.103	0.095	0.100
RMSE	0.541	0.563	0.315	0.340	0.220	0.155	0.292	0.245

**FIGURE 4:**
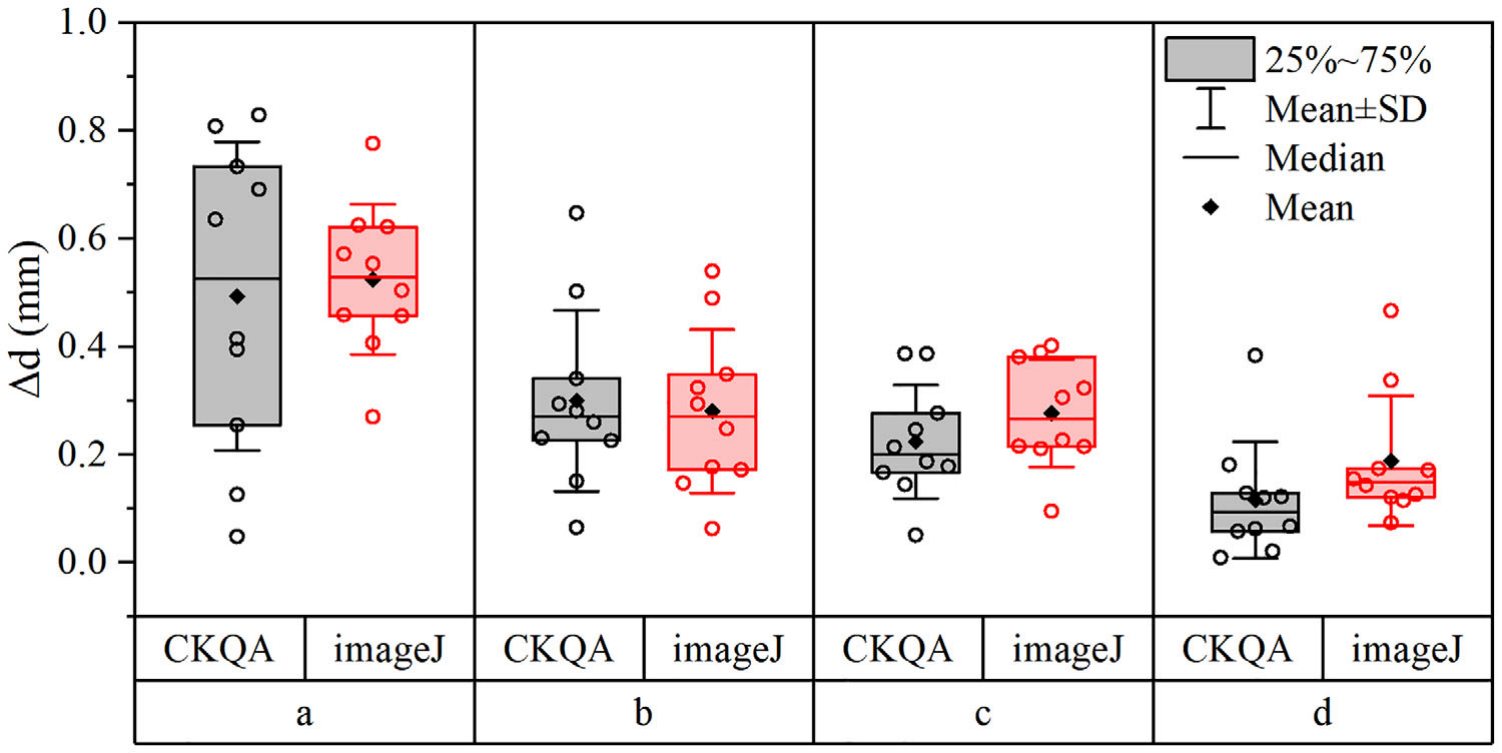
Boxplots of laser‐radiation field coincidence deviations for the eight datasets.

**FIGURE 5:**
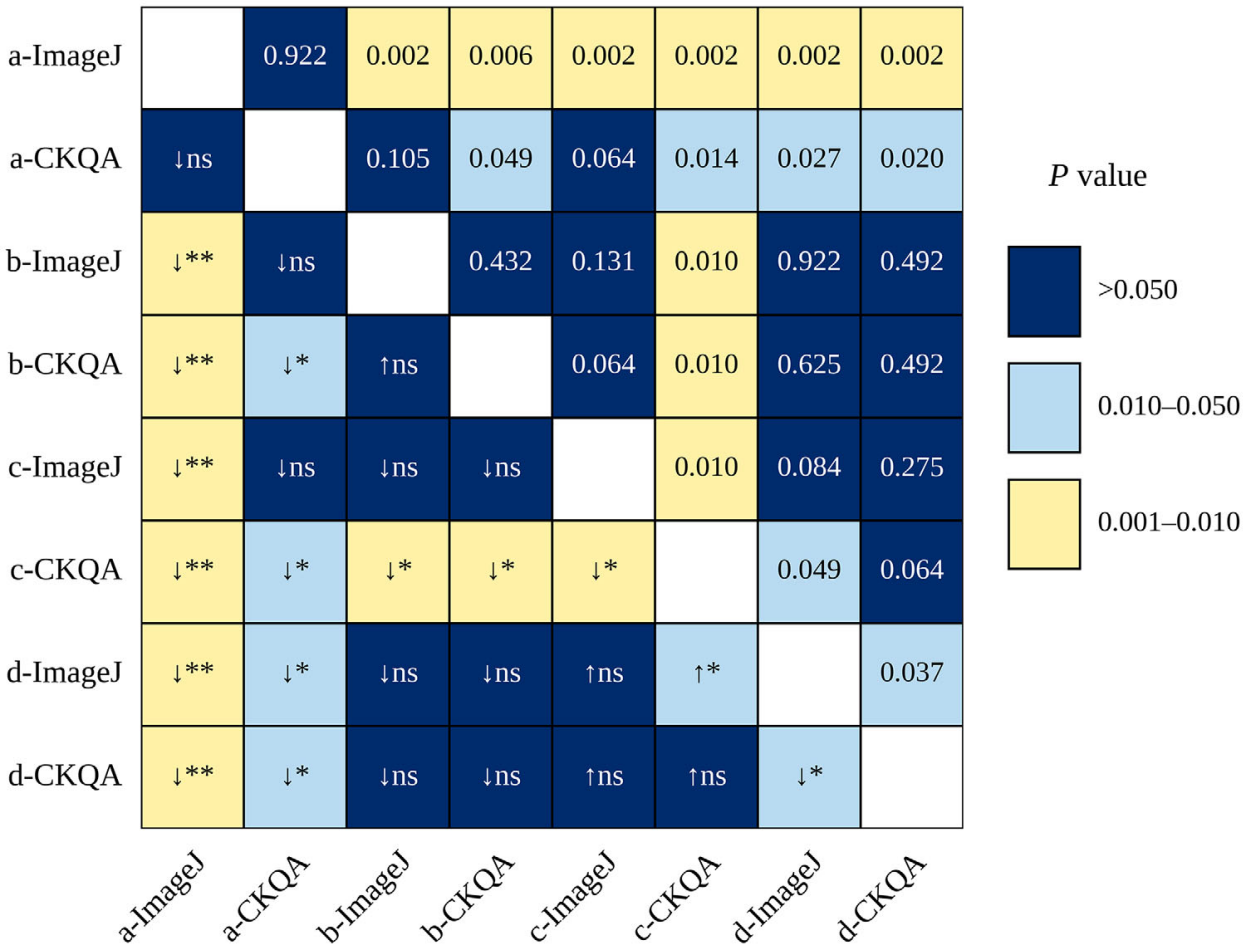
Pairwise comparison matrix across the eight datasets. The upper‐right triangle shows P values from two‐sided exact Wilcoxon signed‐rank tests, and the lower‐left triangle shows the direction and significance of the pairwise difference based on mean deviation (↓ the row dataset has a lower mean deviation than the column dataset; ↑ the row dataset is higher; * *p* < 0.05, ** *p* < 0.01, ns not significant).

### 3.2 Comparison of image‐analysis methods

Within the same measurement workflow, the differences between ImageJ and CKQA depended on the acquisition method. For methods a and b, the two analysis approaches produced similar results, and no statistically significant differences were detected (*p* = 0.922 for a‐ImageJ versus a‐CKQA and *p* = 0.432 for b‐ImageJ versus b‐CKQA). For methods c and d, however, CKQA yielded lower mean deviations, lower standard deviations, and lower RMSE values than ImageJ, with statistically significant differences (*p* = 0.010 for c‐ImageJ versus c‐CKQA and *p* = 0.037 for d‐ImageJ versus d‐CKQA). The contrast was most pronounced for method c, in which the mean deviation decreased from 0.189 mm with ImageJ to 0.116 mm with CKQA.

### 3.3 Effect of collimator diameter

Deviation values remained low overall across the 10 fixed collimator diameters. As shown in Table 1, apart from a few larger values in method a, the measurements were relatively compact within each workflow. No clear monotonic relationship was observed between collimator diameter and deviation, suggesting that within the 5–40 mm range used here, the choice of measurement workflow had a greater influence on the result than collimator size.

## 4 DISCUSSION

The main contribution of this study should be interpreted as practical workflow implementation and preliminary validation rather than as the introduction of a new physical measurement principle or a fundamentally new image‐processing algorithm. Laser‐radiation field coincidence testing has long been included in the established CyberKnife QA framework, including the original AAPM TG‐135 report and the updated TG‐135.B report.^4^, ^5^ The aim of the present study was therefore not to define a new QA item, but to improve the clinical implementation of this established test by reducing manual marking, film handling, scanning, and operator‐dependent analysis. The conventional manual film‐marking workflow is vulnerable to cumulative uncertainty from manual laser marking, direct film handling, fixation, scanning, and subsequent interpretation, which are also consistent with known sources of uncertainty in film‐based QA workflows.^10^, ^11^, ^12^, ^13^, ^14^ The consistently higher deviations observed with method a compared with method b at the same perch position therefore support the practical value of replacing the manual film mark with the central metal marker of the MLC QA tool and allowing alignment through gentle adjustment of the rigid fixture rather than direct manipulation of the film.

An important methodological consideration is that the perch‐based and QASC‐based measurements were acquired at different CyberKnife spatial nodes. This design reflected the practical clinical implementation of the two types of workflows rather than an ideal same‐node comparison. The perch position is convenient for the conventional birdcage‐mounted film workflow described in CyberKnife QA documentation, whereas QASC‐based acquisition requires placement of the detector on the treatment couch because QASC cannot be safely mounted onto the birdcage assembly.^10^, ^25^ Although treatment head alignment was checked with the pinhole laser collimator and the measurement plane was set to an SDD of 800 mm in both setups, node‐dependent robotic mechanical characteristics, including arm extension and potential mechanical sag, may still have contributed to the absolute deviations. This uncertainty is relevant because CyberKnife QA guidance emphasizes geometric consistency, path calibration, and integrated system accuracy.^4^, ^5^, ^8^ Therefore, the lower deviation observed with method c should not be attributed solely to QASC acquisition or CKQA analysis.

For this reason, the most robust interpretation of the present results comes from the within‐node comparisons. At the perch position, methods a and b differed mainly in how the laser reference was defined, and the improvement from method a to method b supports the practical value of replacing manual film marking with the central metal marker of the MLC QA tool within the conventional film‐based QA geometry.^10^ Under the same QASC‐based DailyQA geometry, methods c and d differed mainly in the image‐acquisition medium, and the lower deviation observed with method c supports the practical value of direct digital acquisition compared with film acquisition under otherwise similar setup conditions.^25^ Cross‐node comparisons between perch‐based and DailyQA‐based workflows should therefore be interpreted as pragmatic workflow comparisons rather than as isolated tests of robotic targeting accuracy.

Under the present experimental conditions, method c yielded the lowest measured deviations and RMSE. Importantly, QASC does not natively perform laser‐radiation field coincidence testing. In our workflow, it served only as an external digital image‐acquisition platform. The subsequent deviation measurement was performed offline using ImageJ or CKQA.^25^ Compared with film‐based workflows, direct digital image acquisition reduces film development, scanning, and intermediate handling steps that may introduce additional variability.^10^, ^11^, ^12^, ^13^, ^14^ From an operational perspective, the MLC QA tool also provides a rigid alignment aid: the bright reflection at the central metal tip facilitates visual laser targeting, and the setup can be adjusted by gently moving the tool rather than by pressing directly on a film sheet. These operational advantages were inferred from the workflow structure and were not directly quantified in the present study. The comparison between methods c and d is particularly informative because the geometric setup was essentially identical, and the main difference was the image‐acquisition medium. Therefore, the lower deviations observed with method c support the practical value of a digital acquisition chain under the QASC‐based setup, but should not be interpreted as proof of superior robotic targeting accuracy at a different spatial node.

It should also be emphasized that a small numerical reduction in absolute deviation necessarily translates into a clinically meaningful improvement in treatment accuracy. In the present dataset, all evaluated workflows achieved submillimeter mean deviations and satisfied the commonly used 1.0‐mm clinical tolerance for laser‐radiation field coincidence QA.^4^, ^5^ Therefore, the clinical value of method c should be interpreted primarily from the perspective of workflow robustness rather than from the absolute deviation value alone. Compared with conventional manual film marking, the QASC/CKQA‐based workflow eliminates subjective laser marking, reduces film handling and scanning steps, standardizes image processing and center determination, and may reduce operator‐dependent variability.^10^, ^11^, ^12^, ^13^, ^14^, ^25^ These characteristics are particularly relevant for routine QA programs in which consistency, user independence, and longitudinal trend monitoring are more important than achieving the smallest possible single‐session numerical deviation.

Our results are also consistent with earlier reports from conventional linear accelerator QA. Several EPID‐based or related digital imaging approaches have shown that digital workflows can replace or complement film for light‐radiation field congruence verification while improving efficiency, standardization, and repeatability.^18^, ^19^, ^20^, ^21^, ^22^, ^23^, ^24^ We deliberately avoid equating QASC with a conventional onboard EPID. A more accurate interpretation is that QASC fulfilled a methodologically similar role as a stable external digital image‐acquisition platform, which then enabled more reproducible downstream analysis.^25^

In the analysis comparison, CKQA and ImageJ performed similarly on conventional film‐based images, whereas CKQA yielded lower deviations for the QASC‐based workflows. This finding suggests that automated and standardized image processing may reduce operator‐dependent variability when image quality and boundary definition are favorable. However, this result should not be interpreted as a full validation of CKQA as standalone software. A rigorous software‐validation study requires comparison with mathematical ground truth, for example using synthetic digital phantom images with predefined sub‐pixel laser‐to‐beam offsets. The use of ImageJ as a reference analysis platform is reasonable because it is a widely used image‐analysis tool,^26^ but future validation should evaluate CKQA independently of both ImageJ settings and clinical measurement uncertainty. Not every center has access to QASC because it is a dedicated device with nontrivial purchase cost.^25^ Under those circumstances, method b may represent the most practical routine alternative. Although it remains film based, it avoids at least three major weaknesses of conventional manual film marking: irregular marker geometry, imperfect laser‐marker alignment under glare, and film displacement related to direct pressing or taping after alignment.^10^

The clinical relevance of this work lies in the role of laser‐radiation field coincidence within the broader CyberKnife QA framework. TG‐135 and TG‐135.B explicitly link this item to path calibration, laser alignment checks, and geometric QA, and national or institutional guidance documents include related checks in periodic QA programs.^4^, ^5^, ^6^, ^7^, ^8^, ^9^ From that perspective, our comparison addresses not a new QA principle, but the question of which implementation workflow may be better suited to support a fundamental geometric QA task in routine practice.

The present study was restricted to fixed circular collimators. Although fixed cones provide symmetric radiation fields and relatively straightforward center determination, Iris and InCise MLC fields introduce additional considerations. In particular, MLC fields have rectangular aperture geometry, leaf transmission, different penumbra characteristics, and potentially different requirements for field‐center extraction.^4^, ^5^, ^10^ Our CyberKnife M6 system is not equipped with the Iris collimator, and MLC measurements were not incorporated into the present study because the current study and statistical analysis were based on the 10 fixed circular collimators. Therefore, the conclusions should not be directly generalized to Iris or MLC delivery modes. A dedicated validation study using a larger set of MLC and, where available, Iris apertures will be necessary before this workflow can be recommended for non‐fixed‐cone QA.

Another important consideration is setup repeatability. In the present study, the 10 data points for each workflow were obtained from 10 different fixed circular collimators rather than from repeated setup measurements. Therefore, the observed dispersion reflects aperture‐dependent and workflow‐dependent differences, but it does not directly quantify day‐to‐day setup variance. In routine CyberKnife QA, laser‐radiation field coincidence testing is generally performed as a periodic QA item, such as during quarterly QA, commissioning, or path calibration, and one representative field is commonly selected for a given measurement session.^4^, ^5^, ^10^ Therefore, accumulation of clinically meaningful longitudinal repeatability data requires repeated measurements over a longer observation period. Short‐term repeated measurements within a single session may be useful for estimating immediate setup precision, but they may not fully capture the slow temporal drift of laser‐radiation field coincidence.

Future work may extend the present single‐plane deviation measurement to a multi‐plane beam‐laser geometry model. Such an approach could acquire digital measurements at multiple distances, such as SDD of 800 mm and 1600 mm, to estimate both translational offset and angular misalignment between the CyberKnife central‐axis laser and the MV beam axis.^4^, ^5^, ^8^, ^10^ More importantly, future studies could investigate whether these 3D geometric metrics better predict path‐calibration results or intracranial/extracranial end‐to‐end targeting accuracy than the conventional single‐plane Δd measurement. This question was beyond the scope of the present workflow implementation study and would require dedicated multi‐plane acquisition, controlled perturbation experiments, and correlation with system‐level QA results.

This study has limitations. First, each dataset included only 10 measurements corresponding to 10 fixed collimator sizes; these data should not be interpreted as repeated setup measurements. Second, all experiments were performed on a single CyberKnife M6, so the results should be generalized cautiously. Third, the perch‐based and QASC‐based workflows were performed at different robotic nodes. Although SDD was standardized to 800 mm and treatment head alignment was checked before measurement, the present study cannot fully separate the effects of image‐acquisition medium, analysis software, and node‐dependent robotic mechanics. Fourth, CKQA was not independently validated against synthetic digital phantom images with known mathematical ground truth in the present study. Fifth, the main analysis was restricted to fixed circular collimators; Iris and MLC workflows require dedicated validation before generalization. Sixth, operational benefits such as task time, cost, consumable use, inter‐operator variability, and long‐term reproducibility were inferred from the workflow characteristics but were not directly quantified. Future work should evaluate same‐node comparisons, multi‐user repeatability, longitudinal trend stability, formal time‐and‐motion efficiency, cost‐effectiveness, synthetic‐phantom CKQA validation, field‐specific validation for MLC and Iris apertures, and multi‐plane geometric metrics in conjunction with end‐to‐end testing.^4^, ^5^, ^6^, ^7^, ^8^, ^10^

## 5 CONCLUSIONS

In this CyberKnife M6 study, all evaluated workflows achieved submillimeter mean deviations and met the commonly used 1.0‐mm clinical tolerance for laser‐radiation field coincidence QA. The integrated workflow combining MLC QA tool‐based laser reference definition, QASC‐based external digital image acquisition, and automated CKQA analysis provided a standardized and less operator‐dependent approach for routine laser‐radiation field coincidence QA under the present experimental conditions. The main contribution of this work is practical workflow implementation and preliminary validation rather than the introduction of a new physical measurement principle. In centers where QASC is not available, the film‐based workflow using the MLC QA tool (method b) may serve as a practical alternative because it eliminates manual film marking and reduces alignment‐related handling errors. Future studies should evaluate longitudinal repeatability, inter‐operator variability, operational efficiency, field‐specific validation for MLC/Iris apertures, and multi‐plane 3D beam‐laser geometry metrics.

## CONFLICT OF INTEREST STATEMENT

The authors declare no conflicts of interest.

## CONSENT FOR PUBLICATION

Not applicable.

## ETHICS APPROVAL AND CONSENT TO PARTICIPATE

Not applicable.

## Data Availability

The datasets used and/or analyzed during the current study are available from the corresponding author on reasonable request.
